# Screening of Combinatorial Quality Markers for Natural Products by Metabolomics Coupled With Chemometrics. A Case Study on Pollen Typhae

**DOI:** 10.3389/fphar.2018.00691

**Published:** 2018-06-27

**Authors:** Mingya Ding, Yan Jiang, Xiean Yu, Dong Zhang, Jin Li, Hui Wang, Jiayuan Shen, Xiu-mei Gao, Yan-xu Chang

**Affiliations:** ^1^Tianjin State Key Laboratory of Modern Chinese Medicine, Tianjin University of Traditional Chinese Medicine, Tianjin, China; ^2^Tianjin Key Laboratory of Phytochemistry and Pharmaceutical Analysis, Tianjin University of Traditional Chinese Medicine, Tianjin, China

**Keywords:** natural products, combinatorial quality markers, metabolomics method, chemometric methods, UHPLC-Q-TOF/MS, precise quality evaluation

## Abstract

Natural products, especially for traditional Chinese medicines (TCMs), are of great importance to cure diseases. Yet it was hard to screen the influential quality markers for monitoring the quality. A simple and comprehensive strategy was developed and validated to screen for the combinatorial quality markers for precise quality evaluation and discrimination of natural products. In this study, Pollen Typhae (PT) and it's processed products carbonized PT were selected as the representative case. Firstly, metabolomics data of 49 batches crude PT and carbonized PT was obtained by ultra high-performance liquid chromatography coupled with quadrupole time-of-flight mass spectrometry (UHPLC-Q-TOF/MS). Then, metabolomics approaches were performed to screen for the potential markers that lead to the quality difference. Finally, chemometric methods were used to validate the accuracy of combinatorial quality markers. Thus, 42 compounds were identified from PT, 5 markers (isorhamnetin-3-O-(2^G^-α-L-rhamnosyl)-rutinoside, isorhamnetin-3-O-neohesperidoside, astragalin, kaempferol and umbelliferone) were successfully screened, identified, quantified and regarded as combinatorial quality markers for precise quality evaluation of crude and carbonized PT. It was demonstrated that the established comprehensively strategy provide an efficient tool for precise quality evaluation of natural products from the whole.

## Introduction

Currently, natural products including traditional Chinese medicines (TCMs) gained extensive attention, and be used to cure diseases due to their valuable biological effects including antioxidant, anticancer or antimicrobial activity (Uysal et al., 2017). In contrast to synthetic medicine, many Chinese herbs are commonly subjected to different processing procedures, such as stir-frying, steaming, baking and braising (Wang et al., 2017). Even though TCMs and their related processed products have the same herb source, chemical constituents, pharmaceutical activity and clinical applications of TCMs may vary from different processing procedures (Zhang et al., 2016). For instance, the wine-processed Radix Scutellariae was more effective in cleaning the lung fire, the upper-energizer heat and humidity with the aid of wine characteristic than the crude Radix Scutellariae (Cui et al., 2014). Nowadays, the crude TCMs and their processed products share the same quality specification. It is worth noting that just few constituents of TCMs were regarded as markers for quality evaluation on the basis of Chinese Pharmacopoeia (Wang et al., 2017). However, TCMs were well-known for “multi-chemical components.” Few constituents could not replace all constituents to be the markers for quality evaluation (Yang et al., 2017). Thus, for the sake of precise quality evaluation of crude herbs and its processed products, it is vital to screen for the quality markers according to their effectiveness, practicability and safety.

Typhae Pollen (PT), namely Puhuang, the dry pollen of Typhaceae plants *(Typha angustifolia* L., *Typhae orientalis* Pres or the plants of same genus), was originally recorded in the “Shen Nong Ben Cao Jing.” It was commonly used as herbal medicine to treat dysmenorrhea, stranguria, stroke and angina pectoris (Yu et al., 2017a). The main chemical compositions of PT were flavonoids, sterols, long-chain hydrocarbons, amino acids, organic acids and so on (Qin and Sun, 2005). The pharmacological research indicated that these characteristic constituents have the anti-oxidant, anti-inflammatory, anti-genotoxic, anti-protozoal (Lijun et al., 1998), lowering serum cholesterol (Jia et al., 1990), procoagulantion, anticoagulantion (Gibbs et al., 1983) and hemostasis activities (Chen et al., 2015). PT has been traditionally processed by carbonizing. Modern pharmacological studies have proved that the carbonized PT is more effective in hemostasis (Yan et al., 2017). The reason may be that some certain compounds are enhanced or reduced during processing procedure of PT (Zhou et al., 2016). At present, crude PT and carbonized PT are of commercially availability in the herbal markets. Unfortunately, there is no exact standard to carry out to distinguish PT and carbonized PT. According to Chinese Pharmacopoeia (2015 Version), the total content of isorhamnetin-3-O-(2^G^-α-L-rhamnosyl)-rutinoside and isorhamnetin-3-O-neohesperidoside (not less than 0.5%) is officially set as acceptance criterion for both crude and carbonized PT, which may not be specific and meaningful. Thus, it is urgent to develop a method to discover discriminatory quality markers of PT and carbonized PT.

Chemometrics is a discipline related to the application of mathematics, statistics and computer science (Lavine and Workman, 2008). The main missions of the chemometrics are manipulate and analyze the chemical data, design and choose the optimum measurement procedures to obtain the chemical information to the maximum extent (Kumar et al., 2014). Random forest (RF) and Adaptive boosting algorithm (AdaBoost) are commonly used chemometrics methods, which have been considered as the valuable instrument for data classification and accuracy forecast in application of natural products (Gotoh et al., 2017; Xia et al., 2017). At present, ultra high-performance liquid chromatography tandem mass spectrometry (UHPLC-MS), high performance liquid chromatography tandem mass spectrometry (HPLC-MS) and gas chromatography-mass (GC-MS) have been widely used in the analysis of metabolomics researches (Zhao et al., 2012a,b). Among the above analytical techniques, UHPLC-Q-TOF/MS could provide information more rapidly and efficiently, and allow the wide application for quantitative and qualitative analysis with high selectivity and sensitivity (Yu et al., 2017b; Mocan et al., 2018). Thus, the use of UHPLC-Q-TOF/MS is a powerful method which promotes the development of the field of natural products research, especially for TCMs.

**Figure 1:**
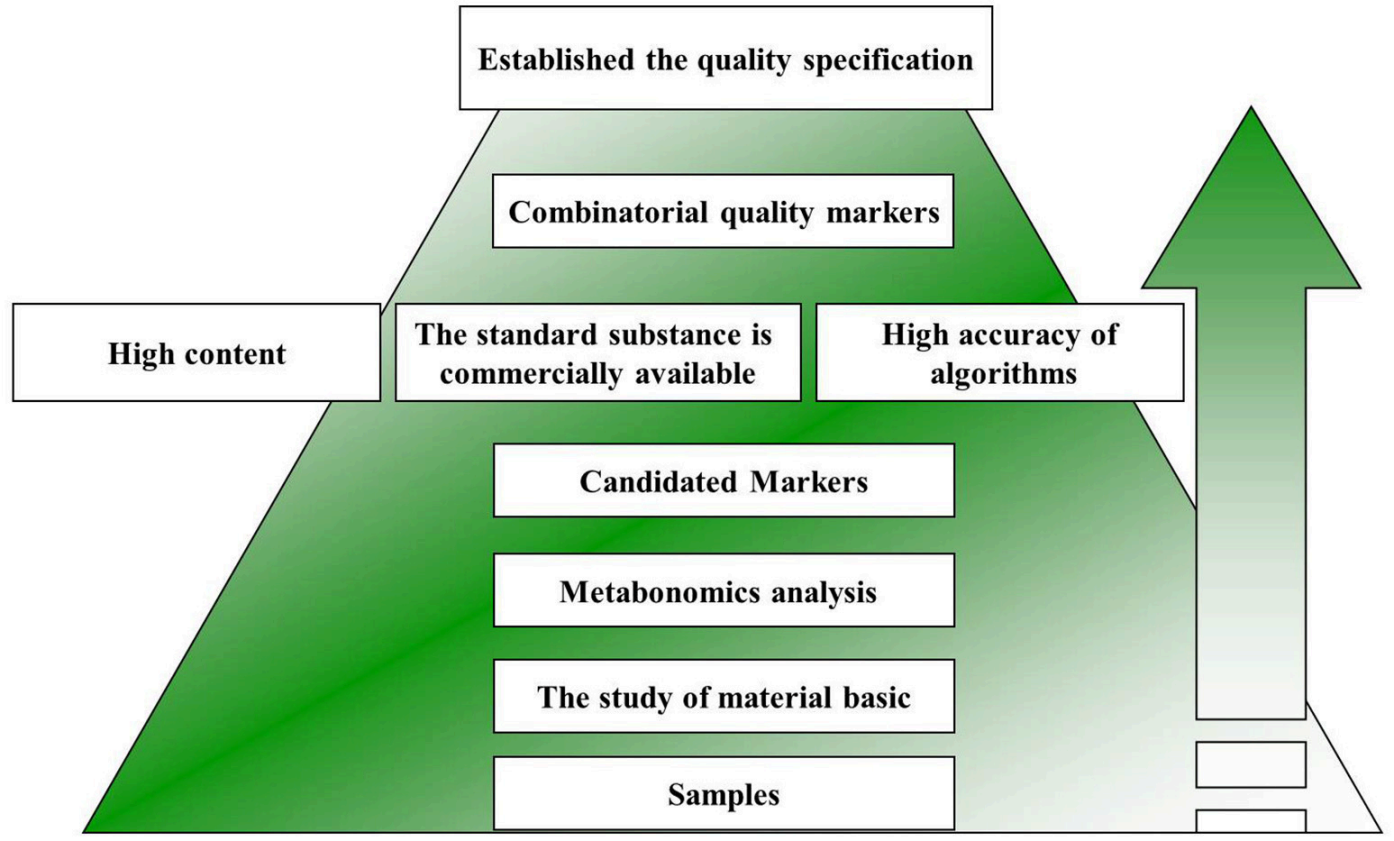
The strategy of screening of combinatorial quality markers for natural products by metabolomics coupled with chemometrics.

The concept of quality markers for quality evaluation of traditional Chinese medicine was first proposed by Changxiao Liu (Yang et al., 2017). In this study, the metabolomics strategy of UHPLC-Q-TOF/MS coupled with chemometrics method was established for discovering the combinational quality markers of natural products. Metabolomics analysis was selected to discover chemical markers which could represent the quality of natural products. Chemometrics models were used to establish a new method for validating the accuracies of the screened markers in natural products. The strategy of the research is summarized in Figure 1. The samples from different producing areas were collected and classified. Then, the chemical constituents were identified by UHPLC-Q-TOF/MS method. Subsequently, the potential markers were screened by metabolomics analysis. Complying with three rules, the final combinatorial markers were identified and screened. First and second rules are that certain potential markers are quantified easily and obtained commercially, respectively. Third rule is that the potential markers could represent the whole chemical information of natural products with high accuracy. Based on the above three rules, these screened combinatorial quality markers were used to establish the quality specification and evaluate the quality of the natural products. Pollen Typhae (PT) was selected as an example. A simple and efficient metabolomics strategy coupled with chemometric was first established and validated to screen for and identify the discriminatory combinatorial quality markers of natural products.

## Materials and methods

### Plant material

A total of 49 batches of Pollen Typhae and carbonized Pollen Typhae (P1-P32 and VP1-VP12 for crude PT, CP1-CP17, and VCP13-VCP22 for carbonized PT) were purchased from different regions of China and authenticated by Dr. Yan-xu Chang (Tianjin University of Traditional Chinese Medicine).

### Chemicals and reagents

Acetonitrile (Dikma Technologies Inc., USA) and methanol (Dikma Technologies Inc., USA) were of HPLC grade. Formic acid of HPLC-grade was purchased from Tedia Company Inc. (Tedia, Fairfield, OH, USA). Deionized water was purified by Milli-Q academic ultra-pure water system (Millipore, Milford, MA, USA). Reference standards such as isorhamnetin-3-O-(2^G^-α-L-rhamnosyl)-ruinosede, umbelliferone, kaempferol, isorhamnetin-3-O-neohesperidoside, astragalin (purity >98%) were purchased from Chengdu Desite Bio-Technology Co., Ltd (Chengdu, China).

### Preparation of sample solutions and reference standards

Preparation of sample solution: 0.500 g of well mixed PT sample powder was accurately weighed and added to 10 mL 70% methanol-water solution. Then the mixture was extracted ultrasonically (40 kHz, 1,200 W power) for 40 min. All the solutions were centrifuged at 14000 rpm and filtered through a 0.22 μm filter membrane before analysis. All solutions were stored at 4°C until use.

Preparation of reference stock standards solution: Five reference standards (isorhamnetin-3-O-(2^G^-α-L-rhamnosyl)-rutinoside, isorhamnetin-3-O-neohesperidoside, astragalin, kaempferol and umbelliferone) were accurately weighed and dissolved in methanol. The mixed stock solution was step-wise diluted to desired concentrations for plotting standard curves.

### UHPLC-PDA analysis

A Waters Acquity UHPLC System (Waters Corp., Milford, MA, USA) consisted of a binary solvent manager, a sampler manager, a column compartment, and a photodiode array (PDA), which was used to perform the quantitative analysis. The quantitative analysis was performed on an ACQUITY UPLC BEH C18 Column (2.1 × 150 mm, 1.7 μm, Waters) at 35°C with a flow rate of 0.3 mL/min. Mobile phase was a mixture of 0.1% formic acid-water (A) and acetonitrile (B). The gradient elution of mobile phase was as follows: 0–2 min, 8–8% B; 2–5 min, 8–18% B; 5–10 min, 18–28% B; 10–12 min, 28–35% B; 12–17 min, 35–70% B; 17–22 min, 70–95% B; 22–27 min, 95–95% B; 27–28 min, 95–98% B. The detection wavelengths were set at 280 nm and the injection volume was 2 μL.

### UHPLC-Q-TOF/MS analysis

Agilent 1290 UPLC system (Agilent Technologies, Waldbronn, Germany) connected to Agilent 6520 Q-TOF mass spectrometer (Agilent Corporation, Santa Clara, CA, USA) via an ESI interface was performed to identify the chemical components in crude PT extract. The gradient program of mobile phase was the same as UHPLC-PDA condition. The optimum operating parameters of Q-TOF/MS were set as follows: drying gas, N_2_; flow rate, 0.3 mL/min; drying gas temperature, 350°C; nebulizer gas pressure, 40 psig; capillary voltage, 3500 V; fragmentor voltage, 120 V; skimmer voltage, 65 V; octopole RF, 750 V; collision energy (CE), 30 and 40 V. Both in positive and negative ion modes, [M-H] ^−^ and [M+H] ^+^ were used to select precursor ions that were subjected to MS/MS analysis. The detection range was m/z 50–1,500 and the UV wavelength was 210–400 nm.

### Method validation

#### Validation of the qualitative method

Validation experiments were performed to investigate the precision, repeatability and stability. P1 sample solution was used for the method validation. The precision was assessed by one sample with six replicate injections. The repeatability of the method was investigated by using six replicate solutions. The stability of those analytes was assessed by analysing the solution at 0, 2, 4, 6, 8, 12, and 24 h. The validation was expressed as the relative standard deviation (RSD).

#### Validation of the quantitative method

##### Calibration curve, limits of detection, and limits of quantification

For the calibration curves, each of the standard stock solutions 5 quantitative compounds was diluted to a different concentration, and each concentration was measured in triplicate. Calibration curves were established by plotting the peak area vs. the concentration of the corresponding analyte solution. The repeatability was tested by six independent samples and expressed as the RSD. The limits of detection (LODs) and limits of quantification (LOQs) were further diluted by the lowest concentration of the mixed standard stock solution to a certain concentration, which were evaluated at a signal-to-noise (S/N) ratio of 3 and 10, respectively.

##### Precision, stability, repeatability, and recovery

The precision of the method was evaluated by intra- and inter-day variations with the six replicates within one day and a repeat of the same steps for three consecutive days, respectively. Six similar samples were extracted and analyzed under the same conditions to measure the repeatability. The sample solution was carried out by 6 replicate injections at 0, 2, 4, 6, 8, 10, 12, 24 h to obtain the stability of the sample. The recovery test was performed by adding the mixed 5 standard solutions to the PT extract samples. All results were evaluated by RSD.

### Data analysis

The metabolomics data in the negative-ionization mode of UHPLC-Q-TOF/MS was imported into the XCMS software operating on the R+ package (R Foundation for Statistical Computing, Vienna, Austria). All detected peaks were tabulated applying tR-m/z pairs and then outputted for statistical analyses. Then, the data of preliminary screening was subjected to partial least-squares discriminant analysis (PLS-DA) by Simca-P (version 14.1, Umetrics, Umea, Sweden). Two algorithms were employed to calculate the accuracy of screened markers by Matlab R2015B (Mathworks, Natick, USA). Finally, the selected combinatorial quality markers were subjected to the Fisher′s discriminant analysis (FDA) for the establishment of the discriminant function by 49 PT samples (P1-P32, CP1-CP17). The FDA was done in SPSS version 19.0 (SPSS, Chicago, IL, USA).

## Results and discussion

### Sample extraction condition optimization

Single factor and orthogonal experiments were effective tools for optimizing the extraction condition in natural products (Cai et al., 2011), which was applied in this experiment to optimize the PT sample extraction condition. The total contents of isorhamnetin-3-O-(2^G^-α-L-rhamnosyl)-rutinoside, isorhamnetin-3-O-neohesperidoside, astragalin, kaempferol and umbelliferone were chosen as the indexes. Single factor tests were performed to obtain a reasonable range of data for the orthogonal experiment. A total of 0.25, 0.5, and 0.75 g PT was added to a 10 mL 70% methanol-water solution, then sonicated (40 kHz, 1,200 W power) at room temperature for 30 min. The results showed that there was higher extraction efficiency by using 0.5 g/10 mL sample/solvent ratio. A total of 0.5 g PT was added to a 10 mL 70% methanol-water solution and sonicated for 30, 45, and 60 min (40 kHz, 1,200 W power). The results indicated that 50 min ultrasonication time produced desirable extraction efficiency. 70, 80, and 90% methanol-water solution was also investigated. 80% methanol-water solution was selected because of the relatively high value obtained. Based on the above results of single factor tests, key factors such as methanol concentration (70, 80, and 90%), sample/solvent ratios (0.25, 0.5, and 0.75 g/10 mL) and ultrasonication time (30, 40, and 50 min) were tested by using an orthogonal L9 (3^4^) experiment. The results indicated that the sample/solvent ratio at 0.5 g/10 mL, 70% methanol-water solution and 40 min extraction time produced desirable extraction efficiency.

### Optimization of chromatographic conditions

**Figure 2:**
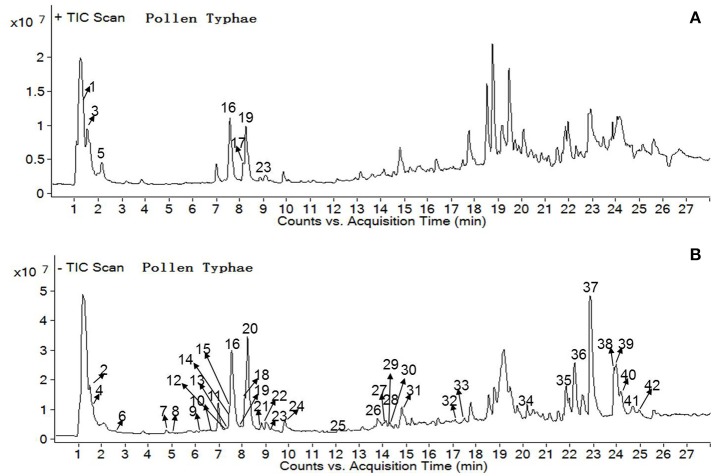
UHPLC-Q-TOF chromatograms of PT sample solution from positive ion mode **(A)** and negative ion mode **(B)**.

**Figure 3:**
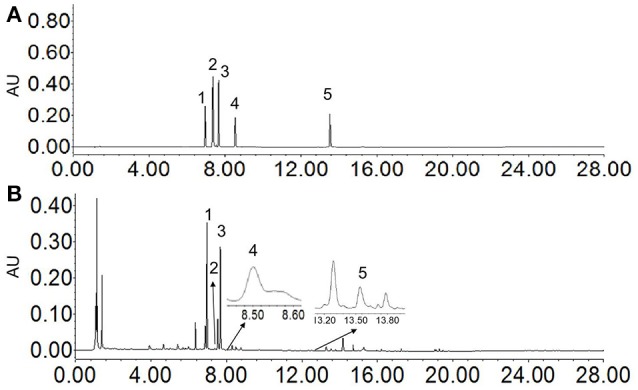
UHPLC chromatograms of standard solution of 5 compounds **(A)** and sample solution **(B)** at 280 nm.

In order to achieve appropriate retention time and optimum resolution of target compounds in natural products, the related factors including the type chromatographic column, the concentration of mobile phase composition, column temperatures, flow rates, and detection wavelengths were investigated. In our UHPLC-PDA analysis method, isorhamnetin-3-O-(2^G^-α-L-rhamnosyl)-rutinoside, isorhamnetin-3-O-neohesperidoside, astragalin, kaempferol and umbelliferone were the target compounds. C_18_ column types (2.1 × 100 mm, 1.7 μm, Waters; 2.1 × 150 mm, 1.7 μm, Waters) and mobile phase's acetonitrile with different aqueous phase (water of 0.1% formic acid or ultra-pure water) were optimized. When the mobile phase was 0.1% aqueous formic acid solution and acetonitrile with C_18_ column (2.1 × 150 mm, 1.7 μm, Waters), the peaks were of high resolution and the peak shapes of the compounds were optimum. In this experiment, PDA detection was used to scan the wavelengths of 200–400 nm, which demonstrated that the 5 compounds all had absorption peaks at 280 nm. The perfect performances were obtained at the flow rate of 0.3 mL/min and column temperature of 35°C. The UHPLC-Q-TOF/MS and UHPLC-PDA chromatograms were shown in Figures 2, 3.

### Method validation

#### Validation of the qualitative method

To validate the developed UHPLC-Q-TOF/MS method, the precision, repeatability and stability of the 11 screened qualitative markers were investigated. The relative standard deviations (RSDs) of precision obtained were less than 4.76%, showing that the method was reliable for the qualitative analysis. The results of the repeatability of the analytes were less than 4.85%, it was demonstrated that the qualitative method was reproducible. The RSDs of the stability were no more than 4.75%, demonstrating the sample solutions were stable for 24 h at room temperature (Table S1). The above results verified that the UHPLC-Q-TOF/MS method could be used for the qualitative investigation of crude PT extract.

#### Validation of the quantitative method

Under the optimum chromatographic conditions, the correlation coefficient values of 5 quantitative components were more than 0.9991. The LOD and LOQ for 5 quantitative components ranged from 0.02 to 0.25 μg/mL and 0.05 to 0.5 μg/mL, respectively. Repeatability was investigated by analysing 6 independent samples. The results indicated that RSD values of repeatability were less than 3.69%. The intra-day and inter-days accuracies for 5 compounds at three levels (low, medium and high) were 86.1–112% and 86.7–112%, respectively. The RSD values of the intra-day and inter-days precisions were 0.19–2.2% and 0.15–3.2%, respectively (Table S2). The remains were 95.8–114% and their RSD values were less than 2.1%, which indicated that the method was of good stability (Table S3). Recovery was performed at low, medium and high levels (accurate adding 80, 100, and 120% of known amount of 5 mixed standards to the analyzed sample of PT). The recovery values of 5 compounds were in the range of 95.8–104% (Table S4), the results demonstrated that the established method was accurate and reliable for the determination of the 5 markers in PT.

### Compound identification in crude PT extract

For the purpose of subsequent screening of discriminatory and combinatorial quality markers in natural products, the study of material basic is very important. Thus, UHPLC-Q-TOF/MS was employed to identify the chemical compounds of natural products. In this study, both positive and negative ion modes were used to obtain more comprehensive information in crude PT extract. The total ion chromatograms (TIC) are shown in Figure 2. As shown in Table 1, 42 compounds were identified or tentatively characterized according to previous reports.

**Table 1:** UHPLC-Q-TOF data and identification of constituents from crude Typhae Pollen extract.

**Peak no**.	**TR**	**Formula**	**Positive ion mode**			**Negative ion mode**			**Identification**	**References**
			**[M+H]^+^**	**MS/MS(+)**	**Δppm**	**[M-H]^−^**	**MS/MS(-)**	**Δppm**		
1	1.257	C_5_H_11_NO_2_	118.0863	118.0866, 72.0815, 55.0553	−0.29	116.0717	–		Valine	García-Salas et al., 2014
2	1.506	C_6_H_8_O_7_	193.0343	–		191.0197	191.0194, 111.0086	0.14	Citric acid	Geng et al., 2014
3	1.578	C_6_H_13_NO_2_	132.1019	132.1023, 86.0971, 69.0709	−2.80	130.0874	–		Leucine	Kivrak et al., 2014
4	1.658	C_4_H_6_O_4_	119.0339	–		117.0193	117.0223, 73.0301	−4.44	Succinic acid	Geng et al., 2014
5	2.120	C_9_H_11_NO_2_	166.0863	166.0861, 120.0808, 103.0544, 91.0701, 77.0392	−1.91	164.0717	–		Phenylalanine	Kivrak et al., 2014
6	2.579	C_12_H_22_O_11_	343.1235	–		341.1089	179.0567, 161.0444, 119.0362, 113.0252	2.579	Sucrose	Wang et al., 2014
7	4.890	C_7_H_6_O_3_	139.0390	–		137.0244	137.0228, 119.0115, 108.0212	4.38	Protocatechuic aldehyde	Xie et al., 2011
8	5.160	C_15_H_14_O_6_	291.0863	–		289.0718	289.0715, 245.0775, 203.0688, 179.0355, 165.0145, 137.0238	2.34	Catechin^a^	Cheng et al., 2011
9	6.311	C_7_H_6_O_2_	123.0441	–		121.0295	121.0290, 77.0402	−4.61	Benzoic acid	Penner et al., 2010
10	6.717	C_16_H_16_O_8_	337.0918	–		335.0772	335.0791, 191.0385, 179.0353, 161.0245, 135.0465	−7.75	Caffeoylshikimic acid	Abu-Reidah et al., 2013; García-Salas et al., 2014
11	6.987	C_33_H_40_O_20_	757.2186	–		755.0240	755.2049, 300.0300, 271.0324, 255.0505, 151.0075	−0.01	Quercetin-3-O-(2^G^-α-L-rhamnosyl)-rutinoside	Tao et al., 2011
12	7.393	C_15_H_14_O_6_	291.0863	–		289.0718	289.0714, 245.0781, 203.0688, 179.0355, 137.0238, 125.0230, 109.0290	1.25	Epicatechin^a^	Cheng et al., 2011
13	7.461	C_9_H_8_O_3_	165.0546	–		163.0401	163.0398, 119.0445	1.63	ρ-Coumaric acid	Fan et al., 2011
14	7.531	C_27_H_30_O_16_	611.1607	–		609.1461	609.1470, 300.0297, 271.0262, 255.0312, 151.0046	−1.46	Quercetin-3-O-neohesperidoside	Tao et al., 2011
15	7.548	C_33_H_40_O_19_	741.2237	–		739.2091	739.2095, 284.0343, 255.0314, 151.0051	−0.54	Kaempferol-3-O-(2^G^-α-L-rhamnosyl)-rutinoside	Tao et al., 2011
16	7.596	C_34_H_42_O_20_	771.2342	771.2360, 625.1261, 479.1192, 317.0662	−2.27	769.2197	769.2202, 314.0456, 285.0442, 151.0049	−0.79	Isorhamnetin-3-O-(2^G^-α-L-rhamnosyl)-rutinoside^a^	Tao et al., 2011
17	7.981	C_9_H_6_O_3_	163.0390	163.0386	2.29	161.0244	–		Umbelliferone^a^	Avula et al., 2016
18	8.140	C_27_H_30_O_15_	595.1657	–		593.1512	593.1532, 284.0345, 255.0319, 151.0045	−0.72	Kaempferol^−^3-O-neohesperidoside	Tao et al., 2011
19	8.275	C_28_H_32_O_16_	625.1763	–		623.1618	623.1634, 314.0451, 285.0422, 151.0005	−2.64	Isorhamnetin-3-O-neohesperidoside^a^	Tao et al., 2011
20	8.493	C_10_H_18_O_5_	219.1277	–		217.1081	217.1098	−7.54	Di-tert-butyl dicarbonate	Ma et al., 2006
21	8.814	C_28_H_32_O_16_	625.1763	–		623.1618	623.1662, 314.0522, 285.0451, 151.0045	−0.77	Isorhamnetin-3-O-rutinoside	Tao et al., 2011
22	9.092	C_21_H_20_O_11_	449.1078	–		447.0971	284.5566, 285.0902	4.51	Astragalin^a^	Tao et al., 2016
23	9.288	C_22_H_22_O_12_	479.1184	479.1206, 317.0697	−4.63	477.1038	477.1057, 314.0448, 285.0434, 151.0055	−3.89	Isorhamnetin-3-O-β-galactoside^a^	Wolfender et al., 2000
24	9.965	C_9_H_16_O_4_	189.1121	–		187.0976	187.0976	0.45	9-hydroxy-nonanoic acid-methyl ester	Ma et al., 2006
25	12.130	C_10_H_18_O_4_	203.1278	–		201.1132	201.1147	4.69	Decanedioic acid	Liu et al., 2012
26	13.960	C_15_H_12_O_5_	273.0757	–		271.0612	271.0625, 151.0046, 119.0509	−4.82	Naringenin^a^	Tao et al., 2011
27	14.025	C_11_H_20_O_4_	217.1434	–		215.1289	215.1302	−4.8	nonanedioic acid-dimethyl ester	Ma et al., 2006
28	14.081	C_15_H_10_O_6_	287.0550	–		285.0405	285.0561	−3.73	Kaempferol^a^	Tao et al., 2011
29	14.179	C_18_H_32_O_5_	329.2323	–		327.2177	327.2176	0.02	Triple hydroxyl-octadecatrienoic acid	Liu et al., 2012
30	14.296	C_16_H_12_O_7_	317.0656	–		315.0510	315.0529, 300.0291, 271.0255, 255.0311	−3.73	Isorhamnetin^a^	Wolfender et al., 2000
31	14.856	C_18_H_34_O_5_	331.2479	–		329.2333	–	0.14	9,10-dihydroxy-Octadecanoicacid-methyl eater	Ma et al., 2006
32	17.358	C_18_H_32_O_4_	315.2373	–		313.2381	–	−0.96	Octadecene dioic acid	Ferreiro-Vera et al., 2013
33	17.495	C_18_H_34_O_4_	315.2530	–		313.2384	–	0.11	Double hydroxyl-octadecatrienoic acid	Ferreiro-Vera et al., 2013
34	20.251	C_18_H_36_O_3_	301.2737	–		299.2592	–	−3.62	12-hydroxystearic acid	Ferreiro-Vera et al., 2013
35	21.807	C_18_H_30_O_2_	279.2319	–		277.2137	–	−4.68	Linolenic acid	Cho et al., 2012
36	22.568	C_16_H_30_O_2_	255.2319	–		253.2173	–	−3.52	9-Hexadecenoic acid	Liu et al., 2012
37	22.890	C_18_H_32_O_2_	281.2475	–		279.2330	–	−3.42	Linoleic acid	Cho et al., 2012
38	23.905	C_16_H_32_O_2_	257.2475	–		255.2330	–	−4.33	Palmitic acid	Ferreiro-Vera et al., 2013
39	23.989	C_18_H_34_O_2_	283.2632	–		281.2486	–	−3.63	Oleic acid	Ferreiro-Vera et al., 2013
40	24.243	C_20_H_36_O_2_	309.2788	–		307.2643	–	−2.82	Palmitic acid ethyl ester	Liu et al., 2012
41	25.681	C_18_H_36_O_2_	285.2788	–		283.2643	–	−2.93	Stearic acid	Cho et al., 2012
42	25.800	C_22_H_40_O_2_	337.3101	–		335.2956	–	−2.07	9,12-Octxdecxdicnoic, butyl ester	Liu et al., 2012

a * confirmed by comparison with reference substances. –Undetected/not comparison with reference data*.

#### Identification of amino acids

Amino acids were commonly known as a class of organic compounds that contain amino and carboxyl groups. HCOOH (46 Da) and NH_3_ (17 Da) moiety were easily generated due to this structure. High polarity determines amino acid has short retention time on the reversed phase column. Benefit from these features, 3 amino acids (Peaks 1, 3, and 5**)** were detected in positive ion mode (Figure S1A; Kivrak et al., 2014).

#### Identification of organic acids

Organic acids play an indispensable role in the treatment of diseases. For example, phenolic acids have the antibacterial and anti-inflammatory effects. Some polyunsaturated fatty acids have the effects of lowering blood lipid and preventing cardiovascular disease (Simopoulos, 1991). Therefore, the identification of this kind of compounds will play a pivotal role in elucidating the mechanism of PT. According to the previous research, organic acids showed good MS response in negative ion mode rather than positive ion mode with the characteristic fragment ions such as CO_2_ (44 Da), CO (28 Da), and H_2_O (18 Da), which helped rapidly identify compounds (Figure S1B; Liu et al., 2016).

Based on the cracking rules deduced above, 6 small phenolic acids (Peaks 2, 4, 7, 9, 10, 13) were tentatively identified with low molecular weight, relatively short retention time and characteristic neutral loss. Peak 2 displayed the molecular formula C_6_H_8_O_7_ at m/z 191 [M-H]^−^. Its characteristic product ions at m/z 111 in MS^2^ spectrum were consistent with the sequential losses of 2H_2_O (36 Da) and CO_2_ (44 Da). By further confirmed the chromatographic and mass spectral information with the reference literature, the compound was easily identified as citric acid (Geng et al., 2014). Peaks 4, 9, and 13 yielded [M-H]^−^ ions at m/z 117, 121, and 163 respectively. They gave the expected fragments at m/z 73, 77, and 119 individually, which were corresponding to the elimination of CO_2_ moiety. Peak 7 displayed [M-H]^−^ ions at m/z 137, and its chemical position deduced as C_7_H_6_O_3_, the MS^2^ ions at m/z 119, 108, which suggested a successive loss of two H_2_O moiety from the parent ion. Further compared their retention behaviors, precise molecular weights and MS/MS spectra information with reported data, peaks 4, 7, 9 and 13 were identified as succinic acid, protocatechuic aldehyde, benzoic acid and ρ-Coumaric acid respectively (Penner et al., 2010; Fan et al., 2011; Xie et al., 2011). Peak 10 exhibited an abundant parent ion [M-H]^−^ at m/z 335, and fragment ions at m/z 191 and m/z 179, which was implies for the presence of quinic acid and caffeic acid moieties. By comparing their chromatographic retention times, mass spectral information and molecular weights with reference data, peak 10 was tentatively identified as caffeoylshikimic acid (Figure S1B) (Abu-Reidah et al., 2013; García-Salas et al., 2014). In addition, 17 fatty acids (peak 20, 24, 25, 27, 29, 31, 32, 33, 34, 35, 36, 37, 38, 39, 40, 41, and 42) were also tentatively identified by comparing with literature data (Ma et al., 2006; Cho et al., 2012; Liu et al., 2012; Ferreiro-Vera et al., 2013).

#### Identification of flavonoids

Flavonoids are considered to be the main bioactive components in PT (Tao et al., 2011). The identification of flavonoids was divided into two steps: identification of flavonoid aglycones and flavonoid glycosides. Flavonoid often exhibit Retro-Diels-Alder (RDA) reactions which are the main feature of the cracking of flavonoids. The C-ring of flavonoids fracture into two parts, ^i,j^A^−^ and ^i,j^B^−^ represent the two parts of the cleavage. i and j are respectively expressed as the position. ^1,3^A^−^ ion as diagnostic ions are frequently appeared in the cleavage fragments of flavonoids. In addition, a few small molecule neutral losses, such as CO (28 Da), CO_2_ (44 Da) and C_2_H_2_O (42 Da) and H_2_O (18 Da), were apt to be formed in consequence of RDA reactions. While the flavonoid glycosides have sugar substituents, which was divided into the fracture of sugar substituents and flavonoid aglycones. Generally, sugar substituents were gradually loss, leaving aglycone ion [${\text{Y}}_{0}^{-}$] or together with radical aglycone anion ([Y_0_-H] ^−^^·^) followed by RDA reactions (Ma et al., 2015). The cleavage sequence could help determine the connect position.

According to the previous research, the flavonoids compounds in PT are mainly quercetin, kaempferol, isorhamnetin and their flavonoid derivatives (Han et al., 2012). The same aglycone ion and/or radical aglycone anion with different abundance at m/z 301/300, m/z 285/284 and m/z 315/314 were usually in negative ion mode after the derivatives of these compounds lose glycosyl groups. However, the compounds that could produce similar ions were not necessarily the same kind of substances. Thus, RDA diagnose fragment ions was needed to further determine and classify the compounds. Glycosyl groups, which attached to the flavonoid aglycones, were usually formed by rhamnosyl (rha, 146 Da), glucosyl (glc, 162 Da) or their combined structure. These described features could be helped for the identification of quercetin, kaempferol, isorhamnetin and their flavonoid derivatives (Ma et al., 2015).

Peak 11 and 14 exhibit [M-H]^−^ at 755 and 609 in negative ion mode, along with the characteristic ion of quercetin at m/z 300 [M-H-rha-glc]^−^ m/z 300 [M-H-rha-glc]^−^ respectively, further confirmed the fragment ions of RDA (m/z 271, m/z 255, m/z 151) and comparison with the reference literature. These two compounds were easily identified as quercetin-3-O-(2^G^−α-L-rhamnosyl)-rutinoside and quercetin-3-O-neohesperidoside (Tao et al., 2011).

Peak 16 and 23 were detected both in the positive and negative ion mode. Compared retention time, mass data and molecular formula mass accuracy (within 5 ppm) with reference literature, peak 22 and 28 were identified as astragalin and kaempferol (Tao et al., 2011, 2016). While peak 15 and 18, which showed [M-H]^−^ at m/z 739 and m/z 593, exhibited radical aglycone anion at m/z 284 [M-H-rha-glc-rha]^−^ and m/z 284 [M-H-glc-rha]^−^, and together with RDA ions at m/z 255 and m/z 151 respectively, indicated that they were flavone derivatives of kaempferol. Further compared their retention behaviors, precise molecular weights and MS/MS spectra information with reported data, peak 15 and 18 were tentatively characterized as kaempferol-3-O-(2^G^−α-L-rhamnosyl)-rutinoside and kaempferol−3-O-neohesperidoside (Tao et al., 2011). Peak 16, 19, 21 and 23 gave the precursor ions [M-H]– at m/z 769, m/z 623, m/z 623, m/z 477 respectively, showed that these peaks were corresponding to the successive loss of glycosyl groups and gave the same fragment ions at m/z 314 ([M-H-rha-glc-rha]^−^, [M-H-glc-rha]^−^, [M-H-glc-rha]^−^, [M-H-glc]^−^), which strongly suggested the presence of glycosyl groups attached to isorhamnetin, subsequently, and RDA ions at m/z 285, m/z 151 were observed. By comparison with literature data, peaks 16, 19, 21 and 23 were identified as isorhamnetin-3-O-(2^G^-α-Lrhamnosyl)-rutinoside, Isorhamnetin-3-O-neohesperidoside, isorhamnetin-3-O-rutinoside and isorhamnetin-3-O-β-galactoside (Mao-chuan et al., 1989; Tao et al., 2011). Peak 30 showed a parent ion at m/z 315 [M-H]^−^, and fragment ions at 300, 271, and 255, corresponding to the mass data of isorhamnetin. (Wolfender et al., 2000).

In addition, Peaks 8 and 12 exhibited the same RDA dissociation with the characteristic RDA ions ^1,3^*A*^−^ (m/z 137), ^1, 4^A^−^ (m/z 125), [M-H-B ring] ^−^ (m/z 179) and ^1, 2^*A*^−^ (m/z 165) fragments in negative ion mode. Some neutral loss fragments like [M-H-CO_2_]^−^ (m/z 245) and [M-H-CO_2_−*C*_2_*H*_2_O]^−^ (m/z 203) were also observed. Compared with literature, they were respectively identified as catechin and epicatechin (Figure S1C; Cheng et al., 2011).

#### Identification of others types

The cracking of the peaks 6 and 17 is not in conformity with the chemical components described above, thus classified as others. Peak 6 displayed [M+HCOOH]^−^ ions at m/z 387. The MS^2^ ions at m/z 179 suggested a glycosyl group cleaved from parent ion. Based on the small molecular weight and the characteristics of glycosyl group, compound 6 was preliminary deduced as glucide, further confirmed by other MS^2^ product ions at 161, 119, 113 with reported literature, peak 6 was tentatively identified as sucrose (Wang et al., 2014). Peak 17 was detected at positive ion mode. Compared with authentic standard substance, it was unambiguously identified as umbelliferone(Figure S1D; Avula et al., 2016). All the information was listed in Table 1.

### Multivariate statistical analysis

Metabonomics analysis was crucial step for screening the combinatorial quality markers in natural products, which was used to screen for the discriminatory markers that could represent the quality of natural products. All chromatographic data of different batches of natural products obtained from UHPLC-Q-TOF/MS was processed by the XCMS software. A three-dimensional data matrices including sample information, variables featured on retention times, m/z value likewise their corresponding intensities were gathered and tabulated (Huang et al., 2013). And then, the *P*-values of *T*-tests were generated and the variables with P values less than 0.05 were chose. Furthermore, Partial least squares discriminant analysis (PLS-DA), a supervised multivariate data analysis technique, characterized by variable selection criteria and potential biomarker output, which was employed to select obviously markers from the above variables with *P*-values less than 0.05. This originates from the truth that it could choose markers counting on variable importance parameters (VIP > 1), then it can be select as markers based on the order of their contributions to the separation of clustering. Thus, the significantly markers with VIP > 1 were selected. In our study, the chromatographic data of P1-P32 and CP1-P17 PT samples was acquired from negative-ionization mode of UHPLC-Q-TOF/MS. A total of 1,091 variables with *P*-values less than 0.05 were obtained. According to the VIP, 311 markers were selected. It was indicated that 311 markers were considered to be the significantly markers which could distinguish the crude PT and carbonized PT.

### Structural study of the selected markers

According to the material basic study of earlier stage for natural products, identification work will continue among the significantly markers with VIP > 1. As for the 311 markers with VIP > 1 of PT samples, 11 compounds were identified as protocatechuic aldehyde, benzoic acid, double hydroxyl-octadecatrienoic acid, kaempferol-3-O-neohesperidoside, ρ-coumaric acid, astragalin, kaempferol, isorhamnetin-3-O-(2^G^-α-L-rhamnosyl)-rutinoside, isorhamnetin-3-O-neohesperidoside, umbelliferone and kaempferol-3-O-(2^G^-α-L-rhamnosyl)-rutinoside in accordance with qualitative research of **3.4**. The remaining markers are still unknown and our identification work will continue.

### Identification of the final dominated combinatorial markers

To explore the final dominated combinatorial quality markers from the above qualitative compounds, three rules were employed. First and second rules are select easily quantified and commercially obtained compounds, respectively. Third rule is that the potential markers could represent the whole chemical information of natural products with high accuracy. Thus, Random Forest (RF) and Adaptive boosting algorithm (AdaBoost), the off-the-shelf supervised learning model, which were introduced for validating the accuracy of the selected markers of every screening procedure. In every model, the half of the samples were set as training set and the remained batches as testing set at random. The training set was used to establish the model and the values of that were regarded as prediction scores. Then, the established model was verified by forecasting the testing set and the accuracy values were gave. In this study, isorhamnetin-3-O-(2^G^-α-L-rhamnosyl)-rutinoside, umbelliferone, kaempferol, isorhamnetin-3-O-neohesperidoside and astragalin were selected from 11 qualitative compounds according to the first and second rules. Then, RF and AdaBoost for categorization and prediction of 49 batches crude PT (P1-P16: training set; P17-P32: testing set) and carbonized PT (CP1-CP8: training set; CP9-CP17: testing set), based on the dataset obtained from the UHPLC-Q/TOF-MS of the 1091, 311, 11 and 5 compounds. The values of accuracy were summarized in Table 2. In every model, the accuracy of 1091, 311, 11, 5 markers were all higher than 90%, It is rather remarkable for the fact that the accuracy of 5 markers were all more than 92%, which approached the accuracy of 1091 variables, Therefore, the 5 markers could replace 1091 variables as combinatorial quality markers for distinguish the crude PT and carbonized PT.

**Table 2:** The values of accuracy of two algorithms.

**Algorithms**	**Different amounts of markers**			
	**1091**	**311**	**11**	**5**
Random forest (RF)	100%	100%	96%	92%
Adaboost	100%	96%	96%	92%

### The quantitative analysis of different batches of PT and its processed product

Quantitative analysis of combinatorial quality markers was a key step in the proposed strategy, which could exhibit the routine variation of natural products and make preparations for establishing the quality specification. The validated UPLC-PDA method was subsequently used to simultaneous determine the 5 combinatorial markers (isorhamnetin-3-O-(2^G^-α-L-rhamnosyl)-rutinoside, isorhamnetin-3-O-neohesperidoside, astragalin, kaempferol and umbelliferone) of 34 batches PT samples (P1-P23 are raw materials, CP1-CP11 for processing products). The results were shown in Table 3. The results showed that there were significant differences between the crude and carbonized PT. The contents of isorhamnetin-3-O-(2^G^-α-L-rhamnosyl)-rutinoside and Isorhamnetin-3-O-neohesperidoside were obviously reduced were after being carbonized, while the contents of astragalin, kaempferol and umbelliferone were slightly reduced. In summary, the contents of 5 components in raw PT were higher than those in processing PT. Thus, this demonstrated that the processing caused the quality fluctuation among crude and carbonized PT samples and probably changed the pharmacological effects, which need to conduct a deep research on this phenomenon.

**Table 3:** The contents of 5 compounds (S1-S5) in 34 batches (P1-P23, CP1-CP11).

	**S1 (%)**	**S2 (%)**	**S3(%)**	**S4 (%)**	**S5 (%)**
P1	0.56	0.0010	0.37	0.00040	0.0010
P2	0.55	0.0010	0.49	0.00070	0.0010
P3	0.36	0.0020	0.33	0.0030	0.0030
P4	0.0020	0.00020	0.00070	–	0.044
P5	0.33	0.013	0.28	0.00030	0.0030
P6	0.32	0.0010	0.31	0.0020	0.0030
P7	0.24	0.00040	0.23	0.0010	0.00050
P8	0.025	0.00030	0.058	0.0010	0.0010
P9	0.57	0.0020	0.52	0.0020	0.0010
P10	0.0005	0.000010	0.0030	–	0.054
P11	0.052	0.0010	0.37	0.00050	0.0010
P12	0.0001	0.0060	0.018	0.00010	0.00050
P13	0.608	0.12	0.54	0.0040	0.0030
P14	0.205	0.00040	0.20	0.00030	0.0030
P15	0.364	0.00050	0.34	0.00030	0.0020
P16	0.193	0.00050	0.19	0.00030	0.0030
P17	0.385	0.0070	0.33	0.00080	0.0020
P18	0.0003	0.0020	0.00020	0.0000080	0.015
P19	0.103	0.00010	0.10	0.00040	0.011
P20	0.079	0.00010	0.067	0.000020	0.057
P21	0.254	0.0010	0.22	0.00070	0.0020
P22	0.48	0.00080	0.55	0.00070	0.0030
P23	0.338	0.0010	0.28	0.00040	0.0020
CP1	0.001	0.0000020	0.00040	0.00050	–
CP2	0.015	0.00010	0.015	0.0010	0.0210
CP3	0.001	–	0.00010	–	0.0010
CP4	0.001	–	0.00030	–	0.0040
CP5	0.0002	–	–	0.00040	0.00030
CP6	0.001	0.0020	0.0010	0.0010	0.00040
CP7	0.001	0.000060	0.00050	–	0.0020
CP8	0.002	–	0.000020	0.0010	0.010
CP9	0.001	0.000030	0.0010	0.00040	0.0010
CP10	0.001	0.000050	0.0010	0.00020	–
CP11	0.001	0.00000070	0.0010	–	0.0010

–* under the limit of quantitation; S1, Isorhamnetin-3-O-(2^G^−α-L-rhamnosyl)-rutinoside; S2, Umbelliferone; S3, = Isorhamnetin-3-O-neohesperidoside; S4, Astragalin; S5, Kaempferol*.

### Discriminant analysis

**Figure 4:**
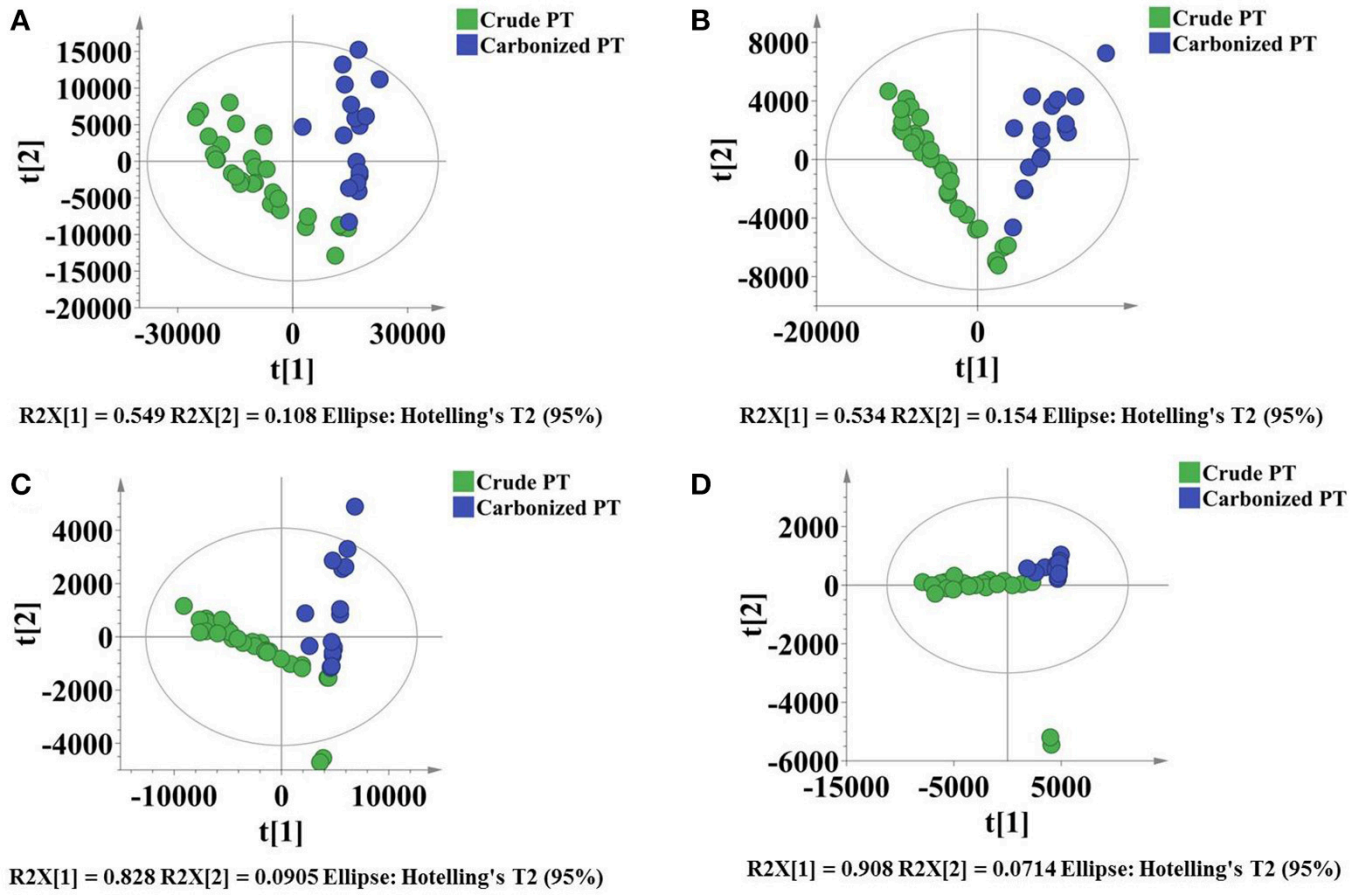
The PLS-DA model for 49 samples with crude PT and carbonized PT depending on 1024 variables **(A)**; 311 markers **(B)**; 11 markers **(C)** and 5 markers **(D)**.

From the perspective of natural products, in particular for crude TCMs and its processing products, the application of discriminant analysis was essential. It could be used to build a predictive model of the group membership based on observed characteristics of the screened combinatorial quality markers of different known samples. It produced a discriminant function. The function could be applied to new samples for rapidly assigning to a group. In our case of PT, a total of 311 markers were selected with VIP > 1 from 1,091 variables, and then 11 markers were identified. Finally, 5 markers were quantified from 11 qualitative markers. Now, 49 batches PT (P1-P32 and CP1-CP17) were prepared with crude PT and carbonized PT, and the PLS-DA model was developed again to validate whether they could be grouped into two parts according to the 1,091, 311, 11 and 5 markers by the dataset obtained from the UHPLC-Q/TOF-MS. Two groups were separated well based on 5 markers in the same way as using 1,091 markers (Figure 4). Therefore, these 5 combinatorial quality markers were subjected to discriminant analysis by SPSS software in order to build predictive model of two groups based on observed features of 49 batches PT (P1-P32 and CP1-CP17). It produced a discriminant function in the unstandardized canonical discriminant analysis model in terms of the predictor variables that offer the distinction among two groups. The discriminant function equation was as follows: γ = −0.000000264573441146107*X*1 + 0.000000246093281974065*X*2 + 0.000006260309339768010*X*3 + 0.000002816986003780560*X*4 − 0.000000260116442845880*X*5 − 0.208119936952103 where X1 to X5 represents the MS response of isorhamnetin-3-O-neohesperidoside, isorhamnetin-3-O-(2^G^-α-L-rhamnosyl)-rutinoside, umbelliferone, kaempferol and astragalin. Eventually, the classification result showed that 95.9% of originally grouped cases were correctly classified and 93.9% of cross-validation grouped cases were further correctly classified. The above results indicated that the discrimination model was reliable. Therefore, an unknown sample can be rapidly assigned to a group by using the discriminant function. The determination value was calculated to be 0.6435 with the value of average of group centroids at −1.458 and 2.745 divided by 2. It indicated that if the discriminant score of sample was lower than the value, the sample will be similar to crude PT sample, or it would be not. In order to validate the accuracy of this predictive model, 22 batches crude PT and carbonized PT samples (VP1-VP12 and VCP1-VCP10) were distinguished by discriminant function (Table S5). According to the discriminant function, PT6 belonged to Group 2, it may due to the contents of different batches vary by Chinese herb's producing areas and processing methods. Meanwhile, the samples of VP1-VP12 and VCP1-VCP10 were successfully divided into two groups respectively, further indicated that the above model was reliable and accurate.

## Conclusion

Along with the increasing demand of natural products for medical care and dietary supplement, the quality control of natural products has been pushed in the spotlight and became a critical issue. In this work, a comprehensive metabolomics coupled with chemometrics strategy was first established and validated to identify the discriminatory combinatorial quality markers for natural products. Take crude and carbonized PT as an example. Five markers [astragalin, kaempferol, umbelliferone, isorhamnetin-3-O-neohesperidoside and isorhamnetin-3-O-(2^G^-α-L-rhamnosyl)-rutinoside] were successfully screened, identified, quantified and regarded as combinatorial quality markers for precise quality evaluation. It was demonstrated that the proposed comprehensive strategy is not only useful in distinguishing crude PT and carbonized PT, but also provide an efficient tool for screening the combinatorial quality markers for other natural products.

## Author contributions

YC, XG, HW, JL, DZ designed the experiment. XY, JS, YJ and MD performed the experiment. MD wrote the manuscript.

### Conflict of interest statement

The authors declare that the research was conducted in the absence of any commercial or financial relationships that could be construed as a potential conflict of interest.
