# Supplementary material for: Screening of Combinatorial Quality Markers for Natural Products by Metabolomics Coupled With Chemometrics. A Case Study on Pollen Typhae
Source: Front Pharmacol. 2018 Jun 27;9:691. doi: 10.3389/fphar.2018.00691 (PMC6033115; doi:10.3389/fphar.2018.00691)
Supplement: Supplementary file 1 [file Table_1.DOCX]

**Table S1** The method validation of UHPLC-Q-TOF/MS

| Compound | Precision(RSD%) | Repeatability(RSD%) | Stability(RSD%) |
| --- | --- | --- | --- |
| Protocatechuic aldehyde | 2.81 | 3.99 | 3.29 |
| Benzoic acid | 0.85 | 0.74 | 2.45 |
| ρ-­Coumaric acid | 1.57 | 1.34 | 1.77 |
| Kaempferol-3-O-(2^G^-α-L-rhamnosyl)-rutinoside | 4.76 | 2.41 | 3.72 |
| Isorhamnetin-3-O-(2^G^-α-L-rhamnosyl)-rutinoside | 3.33 | 2.33 | 3.82 |
| Kaempferol^-^3-O-neohesperidoside | 4.53 | 4.85 | 4.44 |
| Isorhamnetin-3-O-neohesperidoside | 3.05 | 2.74 | 4.15 |
| Astragalin | 4.65 | 4.81 | 4.75 |
| Kaempferol | 1.98 | 1.22 | 3.67 |
| Umbelliferone | 1.20 | 1.75 | 1.77 |
| Double hydroxyl-octadecatrienoic acid | 1.08 | 1.50 | 2.06 |
