# Supplementary material for: Screening of Combinatorial Quality Markers for Natural Products by Metabolomics Coupled With Chemometrics. A Case Study on Pollen Typhae
Source: Front Pharmacol. 2018 Jun 27;9:691. doi: 10.3389/fphar.2018.00691 (PMC6033115; doi:10.3389/fphar.2018.00691)
Supplement: Supplementary file 2 [file Table_2.DOCX]

**Table S2** UHPLC data for the calibration curves, LODs, LOQs and repeatability of the 5 compounds (n = 6)

| Compounds | Regressive equation | Linear range  (μg/mL) | R^2^ | LOD  (μg/mL) | LOQ  (μg/mL) | Repeatability RSD (%) |
| --- | --- | --- | --- | --- | --- | --- |
| Isorhamnetin-3-O-(2^G^-α-L-rhamnosyl)-rutinoside | y=2782.4x+7010.4 | 0.50~600 | 0.9991 | 0.25 | 0.50 | 1.22 |
| Umbelliferone | y=13806x+974.35 | 0.50~100 | 0.9992 | 0.02 | 0.05 | 1.89 |
| Isorhamnetin-3-O-neohesperidoside | y=3988.8x+7965.6 | 0.50~333 | 0.9999 | 0.20 | 0.50 | 1.04 |
| Astragalin | y=8732x+847.5 | 0.5~50 | 0.9999 | 0.10 | 0.30 | 3.69 |
| Kaempferol | y=11513x-613.25 | 0.5~50 | 0.9999 | 0.125 | 0.50 | 1.43 |
