# Supplementary material for: Screening of Combinatorial Quality Markers for Natural Products by Metabolomics Coupled With Chemometrics. A Case Study on Pollen Typhae
Source: Front Pharmacol. 2018 Jun 27;9:691. doi: 10.3389/fphar.2018.00691 (PMC6033115; doi:10.3389/fphar.2018.00691)
Supplement: Supplementary file 3 [file Table_3.DOCX]

**Table S3** UHPLC data for the intra-day, inter-day precision and stability of the 5 compounds (n = 6)

| Compounds | Concentration  (μg/mL) | Intra-day | | Inter-day | | Stability | |
| --- | --- | --- | --- | --- | --- | --- | --- |
|  |  | RSD (%) | Accuracy (%) | RSD (%) | Accuracy (%) | RSD (%) | Remain (%) |
| Isorhamnetin-3-O-(2^G^-α-L-rhamnosyl)-rutinoside | 24 | 1.54 | 105 | 1.20 | 103 | 1.10 | 97.5 |
|  | 120 | 0.31 | 109 | 0.27 | 109 | 0.75 | 110 |
|  | 600 | 1.70 | 112 | 1.10 | 110 | 0.76 | 114 |
| Umbelliferone | 4 | 0.36 | 112 | 0.21 | 112 | 0.23 | 113 |
|  | 20 | 0.22 | 109 | 1.10 | 110 | 0.81 | 114 |
|  | 100 | 0.32 | 98.8 | 0.51 | 98.9 | 1.20 | 111 |
| Isorhamnetin-3-O-neohesperidoside | 13.32 | 0.20 | 86.1 | 0.44 | 86.7 | 1.30 | 104 |
|  | 66.60 | 0.60 | 98.8 | 1.20 | 97.9 | 0.70 | 112 |
|  | 333 | 0.48 | 102 | 1.10 | 101 | 1.20 | 102 |
| Astragalin | 2 | 1.03 | 97.1 | 1.40 | 95.2 | 1.30 | 95.8 |
|  | 10 | 0.54 | 100 | 2.10 | 98.6 | 1 | 96.2 |
|  | 50 | 0.29 | 102 | 0.15 | 101 | 0.60 | 114 |
| Kaempferol | 2 | 2.20 | 101 | 3.20 | 101 | 0.82 | 100 |
|  | 10 | 1.30 | 104 | 2.20 | 107 | 2.10 | 109 |
|  | 50 | 0.19 | 112 | 0.83 | 112 | 0.15 | 111 |
