# Supplementary material for: Screening of Combinatorial Quality Markers for Natural Products by Metabolomics Coupled With Chemometrics. A Case Study on Pollen Typhae
Source: Front Pharmacol. 2018 Jun 27;9:691. doi: 10.3389/fphar.2018.00691 (PMC6033115; doi:10.3389/fphar.2018.00691)
Supplement: Supplementary file 4 [file Table_4.DOCX]

**Table S4** UHPLC data for the recovery of 5 components (n = 6)

| Compounds | Original (μg) | Spiked (μg) | Detected (μg) | Average recovery (%) | RSD (%) |
| --- | --- | --- | --- | --- | --- |
| Isorhamnetin-3-O-(2^G^-α-L-rhamnosyl)-rutinoside | 112 | 90.0 | 201.1 | 99.1 | 0.16 |
|  |  | 112 | 228 | 103 | 1.10 |
|  |  | 134 | 247 | 101 | 1.10 |
| Umbelliferone | 3.30 | 0.26 | 0.58 | 95.8 | 0.56 |
|  |  | 0.33 | 0.67 | 104 | 0.61 |
|  |  | 0.40 | 0.73 | 100 | 0.81 |
| Isorhamnetin-3-O-neohesperidoside | 95.0 | 76.0 | 168 | 96.4 | 0.55 |
|  |  | 95.0 | 189 | 99.0 | 0.57 |
|  |  | 114 | 206 | 97.8 | 1.20 |
| Astragalin | 0.066 | 0.0053 | 0.0713 | 101 | 0.52 |
|  |  | 0.066 | 0.131 | 98.3 | 0.79 |
|  |  | 0.079 | 0.142 | 96.2 | 0.15 |
| Kaempferol | 0.65 | 0.52 | 1.19 | 104 | 0.66 |
|  |  | 0.65 | 1.28 | 98.8 | 0.78 |
|  |  | 0.78 | 1.40 | 96.0 | 0.73 |
