# Supplementary material for: Screening of Combinatorial Quality Markers for Natural Products by Metabolomics Coupled With Chemometrics. A Case Study on Pollen Typhae
Source: Front Pharmacol. 2018 Jun 27;9:691. doi: 10.3389/fphar.2018.00691 (PMC6033115; doi:10.3389/fphar.2018.00691)
Supplement: Supplementary file 5 [file Table_5.DOCX]

**Table S5** The discriminant functions scores of 49 samples

| Sample name | Discriminant score | Classify result | Sample name | Discriminant score | Classify result | Sample name | Discriminant score | Classify result |
| --- | --- | --- | --- | --- | --- | --- | --- | --- |
| P1 | -1.68937201 | 1 | P25 | -1.91584120 | 1 | CP17 | 1.43377844 | 2 |
| P2 | -1.87467450 | 1 | P26 | -2.31831992 | 1 | VP1 | -0.02453385 | 1 |
| P3 | -2.35403268 | 1 | P27 | -2.09187068 | 1 | VP2 | -0.49307292 | 1 |
| P4 | -1.64242256 | 1 | P28 | -2.02276714 | 1 | VP3 | 0.33250387 | 1 |
| P5 | -0.63332590 | 1 | P29 | -1.04487710 | 1 | VP4 | 0.18298637 | 1 |
| P6 | 1.69786697 | 2 | P30 | -1.68772156 | 1 | VP5 | -0.17728478 | 1 |
| P7 | -2.06855831 | 1 | P31 | -2.39221739 | 1 | VP6 | -0.02964979 | 1 |
| P8 | -1.75884498 | 1 | P32 | -1.37665638 | 1 | VP7 | 0.16846878 | 1 |
| P9 | -1.29326715 | 1 | CP1 | 2.25242293 | 2 | VP8 | 0.21294703 | 1 |
| P10 | -1.78946660 | 1 | CP2 | 2.11940702 | 2 | VP9 | -0.26802119 | 1 |
| P11 | -1.62353471 | 1 | CP3 | 4.32905287 | 2 | VP10 | -0.12002615 | 1 |
| P12 | -0.04067996 | 1 | CP4 | 1.17932636 | 2 | VP11 | -0.24687048 | 1 |
| P13 | -1.32438618 | 1 | CP5 | 0.81631856 | 2 | VP12 | -0.40982830 | 1 |
| P14 | -2.09388581 | 1 | CP6 | 2.90814030 | 2 | VCP1 | 4.46329654 | 2 |
| P15 | -0.94569284 | 1 | CP7 | 2.93624837 | 2 | VCP2 | 3.43061244 | 2 |
| P16 | -2.27370838 | 1 | CP8 | 4.38448117 | 2 | VCP3 | 3.26878057 | 2 |
| P17 | -1.10948605 | 1 | CP9 | 1.57228142 | 2 | VCP4 | 3.37493439 | 2 |
| P18 | -0.03006586 | 1 | CP10 | 3.52591200 | 2 | VCP5 | 1.20710952 | 2 |
| P19 | -1.47651148 | 1 | CP11 | 2.74769502 | 2 | VCP6 | 6.01154164 | 2 |
| P20 | -0.96172069 | 1 | CP12 | 2.15525429 | 2 | VCP7 | 0.83732668 | 2 |
| P21 | -1.38437752 | 1 | CP13 | 3.06555287 | 2 | VCP8 | 2.38096915 | 2 |
| P22 | -1.49941148 | 1 | CP14 | 4.63647783 | 2 | VCP9 | 4.08903665 | 2 |
| P23 | -2.12614126 | 1 | CP15 | 3.66342935 | 2 | VCP10 | 5.51563640 | 2 |
| P24 | -1.51178287 | 1 | CP16 | 3.93197538 | 2 |  |  |  |
