# Supplementary figures and images for: Screening of Combinatorial Quality Markers for Natural Products by Metabolomics Coupled With Chemometrics. A Case Study on Pollen Typhae
Source: Front Pharmacol. 2018 Jun 27;9:691. doi: 10.3389/fphar.2018.00691 (PMC6033115; doi:10.3389/fphar.2018.00691)

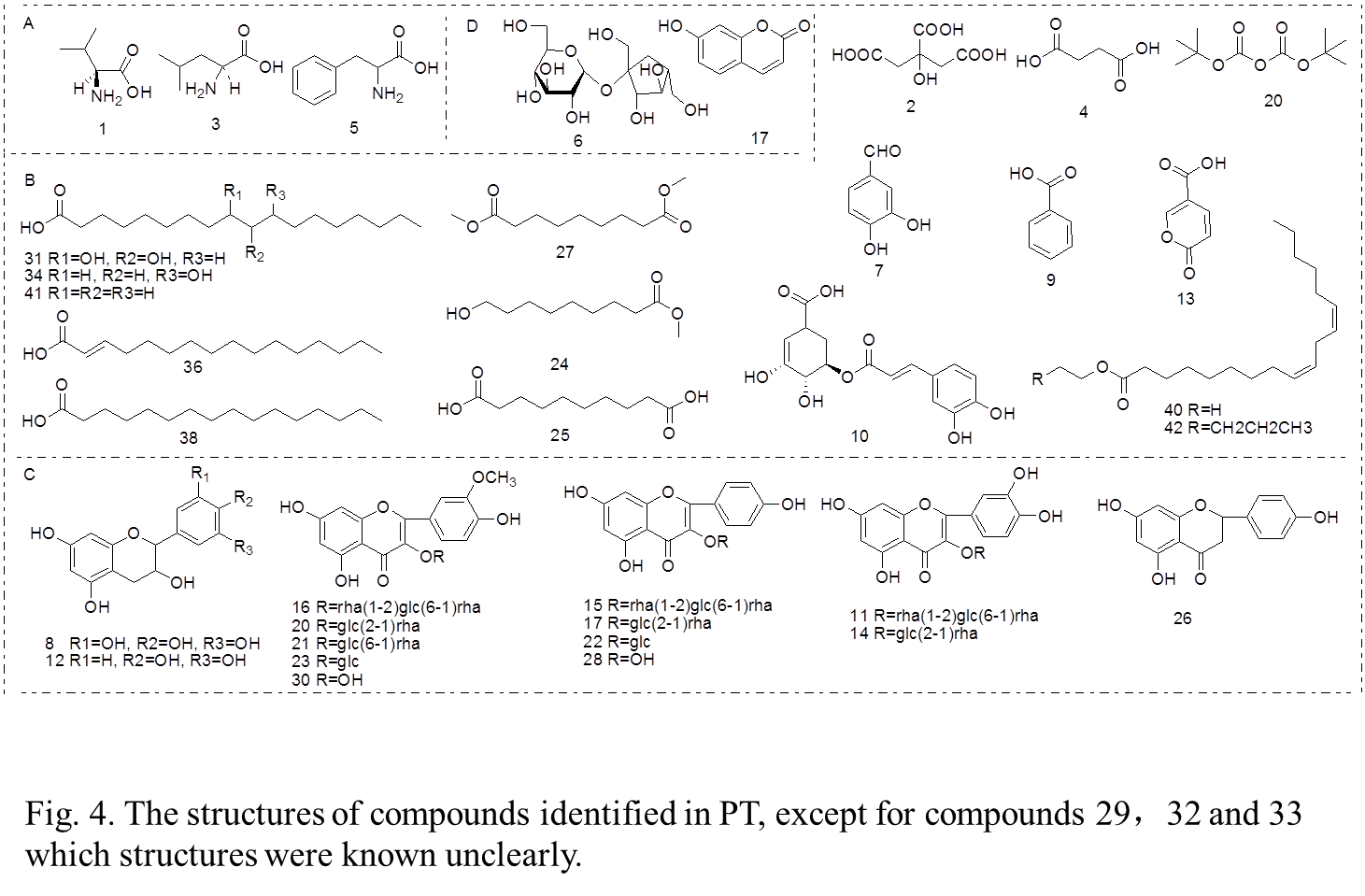

Supplement: Supplementary file 6 [file Image_1.PNG]
